# Ruminal Fiber Degradation Kinetics within and among Warm-Season Annual Grasses as Affected by the Brown Midrib Mutation

**DOI:** 10.3390/ani12192536

**Published:** 2022-09-22

**Authors:** Gonzalo Ferreira, Hailey Galyon, Ayelen I. Silva-Reis, Agustin A. Pereyra, Emily S. Richardson, Christy L. Teets, Phil Blevins, Rebecca R. Cockrum, Matías J. Aguerre

**Affiliations:** 1 School of Animal Sciences, Virginia Tech, Blacksburg, VA 24061, USA; 2 Facultad de Ciencias Agrarias, Universidad Nacional del Nordeste, Corrientes 3400, Argentina; 3 Facultad de Agronomía y Veterinaria, Universidad Nacional de Río Cuarto, Córdoba 5804, Argentina; 4 Virginia Cooperative Extension, Abingdon, VA 24210, USA; 5 Department of Animal and Veterinary Sciences, Clemson University, Clemson, SC 29634, USA

**Keywords:** summer annual forages, brown midrib, fiber digestion kinetics, degradation rate

## Abstract

**Simple Summary:**

Warm-season annual grasses alternative to corn are an attractive option for feeding dairy cattle due to decreased seeding costs and tolerance to drought stress. Warm-season annual grasses include corn, sorghum, and pearl millet. Similar to corn, some varieties of sorghum and pearl millet can contain the brown midrib (BMR) mutation in their genome, which may result in a greater degradability of neutral detergent fiber (NDF). In this study, we evaluated whether forages containing the BMR mutation exhibit the highest NDF digestibility regardless of forage type. For this evaluation, we determined the degradability of NDF by placing forage samples in porous bags within the rumen of lactating dairy cows and measuring how much NDF disappeared over time. Results from this study show that, although forages containing the BMR mutation may show greater NDF degradability than non-BMR forages of the same grass type, forages containing the BMR mutation do not always have the greatest fiber degradability when compared to conventional varieties of other grasses.

**Abstract:**

The objective of this study was to compare the nutritional composition and the neutral detergent fiber (NDF) degradation kinetics of brown midrib (BMR) and non-BMR genotypes within and across warm-season annual grasses. Four commercial varieties (two non-BMR and two BMR) of corn, sorghum, and pearl millet were planted in plots. Forage samples were incubated in the rumen of three rumen-cannulated cows for 0, 3, 6, 12, 24, 48, 96, and 240 h. On an NDF basis, all forage types showed lower acid detergent lignin (ADL) concentrations for BMR genotypes, but the magnitude of the difference differed among forage types. The concentration of undegraded NDF (uNDF; NDF basis) differed among forage types and between genotypes. Corn had the least, pearl millet had the intermediate, and sorghum had the greatest concentration of uNDF. Non-BMR genotypes had greater concentrations of uNDF than BMR genotypes. No interaction existed between forage type and genotype for the concentration of uNDF. In conclusion, although BMR forages may show lower ADL concentrations in the cell wall and greater NDF degradability than non-BMR forages of the same forage type, BMR forages do not always have the least ADL concentration or the greatest NDF degradability when comparing different forage types.

## 1. Introduction

Corn (*Zea mays* (L.)), in the form of whole-plant corn silage, is a major component of diets for high-producing dairy cows. To most dairy farmers, the great biomass production per unit of land and the quality of the biomass produced make of whole-plant corn silage an attractive feed ingredient to include in diets of dairy cattle. However, different factors linked to whole-plant corn silage, such as the high cost of the seed, the lack of fit in the crop rotation [1], or the poor tolerance to drought [2,3,4], motivate some producers to seek alternative forages.

Warm-season annual grasses alternative to corn are an attractive option to dairy producers. These warm-season annual grasses include forage sorghum (*Sorghum bicolor* (L.) Moench), Sudan grass (*Sorghum sudanense* (Piper) Stapf), the hybrids of sorghum and Sudan grass, or pearl millet (*Pennisetum glaucum* (L.)). Similar to corn, some varieties of these forages can contain the brown midrib (BMR) mutation in their genome, which may result in a greater neutral detergent fiber (NDF) degradability (NDFD) [5]. For example, Oba and Allen reported greater 30-h in vitro NDFD for BMR corn silage than for non-BMR corn silage (55.9 vs. 46.5% NDFD) [6]. Similarly, Oliver et al. reported greater 48-h in situ NDFD for diets containing BMR sorghum silage than for diets containing non-BMR sorghum silage (61.7 vs. 56.4% NDFD) [5], and Mustafa et al. reported greater 96-h in situ NDFD for BMR pearl millet fresh forage than for non-BMR pearl millet fresh forage (78.4 vs. 64.4% NDFD) [7].

Despite the presence of the BMR mutation consistently demonstrating greater NDFD, grasses with the BMR mutation might not always have the greatest NDFD. Yang et al. fed high-producing dairy cows with diets containing either a non-BMR corn silage or a BMR sorghum silage, and observed a similar (56.7%) total tract NDFD [8]. These data suggest that the simple fact of containing the BMR mutation does not ensure the greatest NDFD among forages. Furthermore, Yang et al. reported that BMR sorghum silage contained 11.6% acid detergent lignin (ADL; NDF basis), whereas the non-BMR corn silage contained 5.8% ADL (NDF basis) [8]. This observation indicates that forages containing the BMR mutation do not necessarily have the lowest ADL concentration (NDF basis) when comparing forage types. Interestingly, the study of Yang et al. is not the first to show this [8]. For example, Bernard and Tao [9] reported 8.3 and 14.2% ADL concentration (NDF basis) for non-BMR corn silage and BMR sorghum silage, respectively. In other studies, however, the difference for ADL concentration in the NDF fraction between non-BMR corn silage and BMR sorghum silage was minimal (i.e., less than 2% units) [10,11,12]. All these observations suggest that, when comparing different forage types, those carrying the BMR mutation do not always have the lowest ADL concentration in the NDF fraction.

The hypothesis of this study is that warm-season annual grasses containing the BMR mutation have lower ADL concentrations and greater NDF degradability than non-BMR warm-season annual grasses when compared within forage type but not across forage types. Therefore, the objective of this study was to compare the nutritional composition and NDF degradation kinetics of BMR and non-BMR genotypes within and across types of warm-season annual grasses.

## 2. Materials and Methods

### 2.1. Seed Materials and Locations

Four commercial varieties of corn, sorghum, and pearl millet were planted in triplicate in small plots (1.5 m × 3.0 m) to obtain whole-plant and tissue samples. All seeds were obtained based on commercial availability and, therefore, were randomly selected. For each of the three grass types, two non-BMR and two BMR varieties were planted. Corn varieties included A5262GT3D (non-BMR; Augusta Seeds, Verona, VA, USA), BMR14B96 (BMR; Mycogen Seeds, Indianapolis, IN, USA), TMF14R77 (non-BMR; Mycogen Seeds), and P1449AMX (BMR; Pioneer Hi-Bred International, Johnston, IA, USA). Sorghum varieties included: AF 8301 (non-BMR; Alta Seeds, Amarillo, TX, USA), ADV F7232 (BMR; Alta Seeds), Green Graze Supreme (non-BMR; Green Seed Company, Springfield, MO, USA), and SS130 (BMR; Southern States Cooperative, Richmond, VA, USA). Pearl millet varieties included: Wonderleaf (non-BMR; Alta Seeds), Prime 180 (BMR; King’s AgriSeeds, Lancaster, PA, USA), SS635 (non-BMR; Coleman Farm Supply, Appomattox, VA, USA), and Prime 360 (BMR; King’s AgriSeeds).

All varieties were planted at two locations (Blacksburg and Glade Spring, VA, USA) in two growing seasons (2019 and 2020). To avoid confounding effects between forage quality and growing conditions (e.g., planting date and growing degree days), all grasses were planted on the same day within a site and season. In 2019, plots were planted on 30 May in Blacksburg and 28 June in Glade Spring. In 2020, plots were planted on 15 June in Glade Spring and on 20 June in Blacksburg. The soils were classified (web soil survey; www.nrcs.usda.gov; accessed on 21 September 2022) as Hayter loam with a land capability classification of IIe for Blacksburg and as Wyrick–Marbie complex silt loam with a land capability classification of IIIe for Glade Spring. 

### 2.2. Sample Collection and Analyses

To avoid confounding effects between forage quality and growing conditions (e.g., harvesting date and growing degree days), all grasses were harvested on the same day within a site and season. In 2019, plants and plant tissues were collected on 4 September in Blacksburg and on 30 September in Glade Spring. In 2020, plants and plant tissues were collected on 16 September in Blacksburg and on 30 September in Glade Spring. Harvesting occurred when the kernels of corn plants had ¼ to ½ milk lines [13]. Concurrently, sorghum and pearl millet grains were between the milky and soft dough stages.

At harvesting time, five plants from two randomly selected spots within each plot (i.e., 10 plants per plot) were cut by hand at approximately 15 cm above ground. Whole plants were chopped with a Stanley CH2 wood chipper (GXi Outdoor Power, LLC, Clayton, NC, USA). After mixing thoroughly within a barrel, a sample of the chopped material was placed in a plastic bag, immediately placed in a cooler with dry ice, and transferred to the laboratory for storage at −20 °C. In addition, 5 to 10 plants from each plot were dissected by hand into stem internodes and leaf blades, and all stem internodes and leaf blades from the whole plant were placed in a bag, immediately placed in a cooler with dry ice, and transferred to the laboratory for storage at −20 °C.

Chopped whole-plant and tissue samples were thawed and dried at 55 °C in a forced-air oven (Freas 645, Thermo Electron Corporation, Marietta, OH, USA) until constant weight was obtained. Then, samples were ground to pass through a 1-mm screen of a Wiley mill (Thomas Scientific, Swedesboro, NJ, USA). Crude protein (CP) concentration was calculated as percent N × 6.25 after combustion analysis (Method 990.03, AOAC, 2019) [14] using a Vario El Cube CN analyzer (Elementar Americas, Inc., Mount Laurel, NJ, USA). Ash-free neutral detergent fiber (NDF) and acid detergent fiber (ADF) concentrations were determined using the Ankom200 Fiber Analyzer (Ankom Technology, Macedon, NY, USA). Sodium sulfite and α-amylase (Ankom Technology) were included for NDF analysis [15]. Acid detergent fiber and acid detergent lignin (ADL) concentrations were determined sequentially. After determining ADF weights, residues were incubated for 3 h in 72% sulfuric acid within a 4-L jar that was placed in a DaisyII Incubator (Ankom Technology).

### 2.3. Ruminal In Situ NDF Degradability

Ground samples (0.25 g) were inserted into acetone-rinsed porous bags (F57, Ankom Technology) and incubated (9:00 am) in the rumen of three rumen-cannulated and lactating cows fed a total mixed ration containing 42% corn silage, 5% triticale silage, 3% mixed grass hay, and 50% concentrate mix (DM basis). Bags were extracted from the rumen 0, 3, 6, 12, 24, 48, 96, and 240 h after initial incubation, rinsed three times (3-min washing cycles) using a washing machine (SKY2767, Best Choice Products, Irvine, CA, USA), and dried in a forced-air oven at 55 °C for 48 h. After weighing, all bags were extracted in neutral detergent with α-amylase and sodium sulfite [15], reweighed, and combusted to determine the residual ash-free NDF.

Degradation kinetic parameters were estimated using the NLIN procedure of SAS (SAS version 9.4, SAS Institute Inc., Cary, NC, USA). Ruminal in situ NDF degradability (ISNDFD) was determined as

*ISNDFD* = [(100 − *uNDF*) × (1 − *e*^(*−kd*×*T*)^)]

where ISNDFD is the degraded NDF (% NDF) at time of fermentation T (h), uNDF (% NDF) is the undegraded NDF after 240 h of fermentation, and kd is the degradation rate (%/h) of the potentially degradable NDF (pdNDF; % NDF). The soluble fraction of NDF (commonly known as fraction a) was assumed to be 0 as NDF is not immediately degradable in the rumen [16] and no lag time was assumed [17]. 

The effective ruminal degradability (ERD) of NDF (% NDF) was determined as

*ERD = pdNDF ×* [*kd/(kd + kp)*]

where pdNDF is the concentration of pdNDF on an NDF basis (% NDF) and kp is the passage rate at 4%/h [18].

### 2.4. Statistical Analysis

The experiment was designed as a completely randomized design with spatial and temporal replication (i.e., two sites and two seasons). All variables were analyzed using the MIXED procedure of SAS (SAS version 9.4, SAS Institute Inc., Cary, NC, USA). The statistical model included the fixed effect of year (degrees of freedom (df) = 1), the fixed effect of location (df = 1), the fixed effect of forage type (df = 2), the fixed effect of genotype (fixed; df = 1), all two-, three-, and four-way interactions (df = 18), and the random residual error (df = 120). Statistically significant differences were declared at *p* ≤ 0.05, and tendencies to statistically differ were declared at *p* ≤ 0.10.

## 3. Results

### 3.1. Plant and Tissue Composition

For whole plants (Table 1), the concentration of CP differed among forage type (*p* < 0.01) and genotype (*p* = 0.02), but no interaction existed. Pearl millet had the greatest concentration of CP (8.8% CP), sorghum had the intermediate concentration of CP (7.3% CP), and corn had the lowest concentration of CP (6.1% CP). Plants with the BMR genotype had a greater CP concentration than non-BMR plants, but only differed by 0.4% units (7.6% vs. 7.2% CP). The concentration of NDF differed among forage types (*p* < 0.01) but did not differ between non-BMR and BMR genotypes (*p* = 0.89). Corn had the lowest concentration of NDF (42.3% NDF), while sorghum and pearl millet had the greatest concentrations of NDF (49.0 and 50.4% NDF, respectively). No interaction existed between forage type and genotype for NDF concentration. The concentration of ADF differed among forage types (*p* < 0.01) being lowest for corn (23.4% ADF) and greatest for sorghum and pearl millet (30.3 and 29.2% ADF, respectively). An interaction tended to exist between forage type and genotype for ADF concentration (*p* = 0.10). This interaction is attributed to the lower ADF concentration of BMR genotypes observed in corn (24.1 vs. 22.6% ADF) and pearl millet (29.8 vs. 28.5% ADF), but not in sorghum (30.1 vs. 30.4% ADF). On a DM basis, an interaction existed between forage type and genotype for ADL concentration (*p* < 0.01). All forage types showed lower ADL concentrations for BMR genotypes, but the magnitude of the difference differed, being 1.1% units for pearl millet, 0.7% units for corn, and 0.4% units for sorghum. Corn (1.9% ADL), pearl millet (3.2% ADL), and sorghum (4.1% ADL) had the lowest, intermediate, and greatest ADL concentrations on a DM basis, respectively. On an NDF basis, an interaction existed between forage type and genotype for ADL concentration (*p* = 0.02). All forage types showed lower ADL concentrations for BMR genotypes, but the magnitude of the difference differed, being 2.1% units for pearl millet, 1.4% units for corn, and 0.9% units for sorghum. Corn had the lowest concentration of ADL on an NDF basis (5.0 and 3.6% ADL for non-BMR and BMR, respectively), whereas sorghum had the greatest concentration of ADL on an NDF basis (8.7 and 7.8% ADL for non-BMR and BMR, respectively). Pearl millet had an intermediate concentration of ADL on an NDF basis (7.3 and 5.2% ADL for non-BMR and BMR, respectively).

For leaf blades (Table 1), the concentration of CP differed among forage types (*p* < 0.01) but did not differ among genotypes. Corn had the lowest (11.6% CP), pearl millet had intermediate (12.2% CP), and sorghum had the greatest (13.7% CP) concentration of CP. No interaction existed between forage type and genotype for CP concentration. The concentration of NDF differed among forage types (*p* < 0.01), but did not between non-BMR and BMR genotypes (*p* = 0.75). Leaf blades from pearl millet had the lowest concentration of NDF (55.8% NDF), while leaf blades from corn and sorghum had similar concentrations of NDF (59.2 and 59.8% NDF, respectively). No interaction existed between forage type and genotype for NDF concentration. The concentration of ADF differed among forage types (*p* = 0.02), being lowest for pearl millet (31.5% ADF) and greatest for corn and sorghum (34.2 and 33.8% ADF, respectively). The concentration of ADF did not differ between genotypes (*p* = 0.12) and no interaction existed (*p* = 0.93) between forage type and genotype for ADF concentration. On a DM basis, an interaction existed between forage type and genotype for ADL concentration (*p* = 0.04). Corn and pearl millet showed lower ADL concentrations for BMR genotypes, but the magnitude of the difference differed, being 1.4% units for pearl millet and 1.0% units for corn. The concentration of ADL did not differ between genotypes for sorghum (4.2% ADL). On an NDF basis, an interaction existed between forage type and genotype for ADL concentration (*p* = 0.02). Corn and pearl millet showed lower ADL concentrations for BMR than for non-BMR genotypes, but the magnitude of the difference differed, being 2.8% units for pearl millet and 1.7% units for corn. The concentration of ADL did not differ between genotypes for sorghum (7.0% ADL). The concentration of ADL on an NDF basis did not differ among forage types (7.0% ADL).

For stem internodes (Table 1), the concentration of CP differed between forage types (*p* < 0.01) and genotypes (*p* < 0.01). Corn had the lowest CP concentration (1.8% CP), while sorghum and pearl millet had the greatest (2.4% and 2.6% CP, respectively). Non-BMR genotypes had a lower CP concentration (2.0% CP) than BMR genotypes (2.5% CP). An interaction did not exist between forage type and genotype for CP concentration in stem internodes. An interaction existed between forage type and genotype for NDF concentration (*p* < 0.01). Corn and pearl millet showed lower NDF concentrations for BMR than for non-BMR genotypes, but the magnitude of the difference differed, being 11.7% units for corn and 8.3% units for pearl millet. Non-BMR and BMR genotypes had similar concentrations of NDF for sorghum (54.2% NDF). An interaction existed between forage type and genotype for ADF concentration (*p* < 0.01). Corn and pearl millet showed lower ADF concentrations for BMR than for non-BMR genotypes, but the magnitude of the difference differed, being 9.9% units for corn and 6.1% units for pearl millet. Non-BMR and BMR genotypes had similar concentrations of ADF for sorghum (37.0% ADF). Concentrations of ADF were similar among forage types (36.2% ADF). On a DM basis, an interaction existed between forage type and genotype for ADL concentration (*p* < 0.01). Non-BMR genotypes had greater concentrations of ADL than BMR genotypes in all forage types (*p* < 0.01), although the magnitude of the difference differed among forage types, being 2.6% units for corn, 2.7% units for pearl millet, and 0.7% units for sorghum. The concentration of ADL differed among forage types (*p* < 0.01), such that sorghum had the greatest concentration (4.5% ADL) and corn had the least (3.5% ADL). On an NDF basis, an interaction existed between forage type and genotype for ADL concentration (*p* < 0.01). Non-BMR genotypes had greater concentrations of ADL than BMR genotypes in all forage types (*p* < 0.01), although the magnitude of the difference differed among forage types, being 3.8% units for pearl millet, 3.6% units for corn, and 1.5% units for sorghum. The concentration of ADL differed among forage types (*p* < 0.01), such that sorghum had the greatest ADL concentration (7.9% ADL), pearl millet had the intermediate (6.8% ADL), and corn had the lowest (6.2% ADL).

### 3.2. Fiber Degradation Kinetics

**Figure 1:**
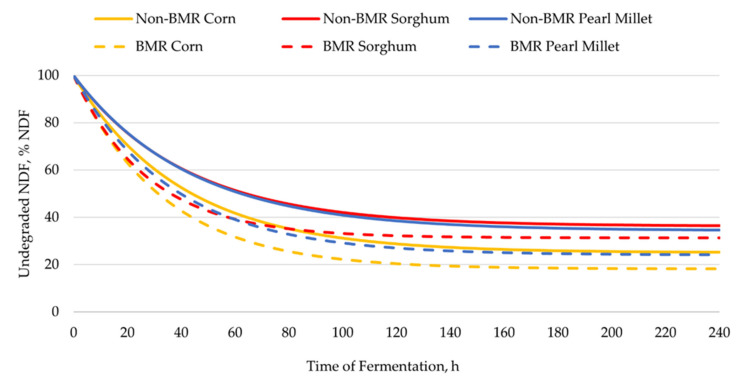
Degradation kinetics of whole plants of warm-season annual grasses of different forage types (corn, sorghum, or pearl millet) and genotypes (brown midrib (BMR) or non-BMR). Each curve represents the residual or undegraded neutral detergent fiber (uNDF) of each forage type by genotype combination that remained after its ruminal fermentation.

For whole plants (Table 2; Figure 1), the concentration of uNDF (NDF basis) differed among forage types (*p* < 0.01) and between genotypes (*p* < 0.01), but an interaction did not exist. On an NDF basis, corn had the lowest concentration of uNDF (21.7% uNDF), pearl millet had an intermediate concentration of uNDF (29.2% uNDF), and sorghum had the greatest concentration of uNDF (33.8% uNDF). Non-BMR genotypes had greater (*p* < 0.01) concentrations of uNDF (31.9 vs. 24.5% uNDF; NDF basis) than BMR genotypes. Because uNDF and pdNDF are complementary percentages, corn had the greatest concentration of pdNDF (78.3% pdNDF), pearl millet had an intermediate concentration of pdNDF (70.8% pdNDF), and sorghum had the lowest concentration of pdNDF (66.2% pdNDF). Similarly, BMR genotypes had 7.4% units more pdNDF (NDF basis). The degradation rate of pdNDF did not differ among forage types, whereas BMR genotypes had a faster degradation rate of pdNDF than non-BMR genotypes in all forage types (3.1%/h vs. 2.4%/h; *p* < 0.01). Corn had a greater (*p* < 0.01) ERD of NDF (31.5% NDF) than sorghum and pearl millet (26.9% NDF). Brown midrib genotypes had greater (*p* < 0.01) ERD of NDF (31.6% NDF) than non-BMR genotypes (25.3% NDF). No interaction existed between forage type and genotype for ERD of NDF.

For leaf blades (Table 2), an interaction existed between forage type and genotype (*p* = 0.02) for uNDF concentration (NFD basis). Sorghum had the greatest concentration of uNDF (21.7% uNDF), corn had an intermediate concentration of uNDF (18.0% uNDF), and pearl millet had the lowest concentration of uNDF (16.0% uNDF). Non-BMR genotypes contained greater concentrations of uNDF than BMR genotypes in corn (21.4 vs. 14.5% uNDF) and sorghum (23.3 vs. 20.2% uNDF), but not in pearl millet (16.0% uNDF). All forage types had similar degradation rates of pdNDF (2.8%/h; *p* = 0.86), and BMR genotypes tended to have greater degradation rates of pdNDF than non-BMR genotypes (2.9 vs. 2.7%/h, respectively; *p* = 0.08). No interaction existed between forage type and genotype for the degradation rates of pdNDF. All forage types had similar ERD of NDF (32.7% NDF), and BMR genotypes had greater ERD of NDF than non-BMR genotypes (34.2 vs. 31.2% NDF, respectively; *p* = 0.02). No interaction existed between forage type and genotype for the ERD of NDF. 

For stem internodes (Table 2), an interaction existed between forage type and genotype (*p* = 0.02) for uNDF concentration (NFD basis). Among forage types, the concentrations of uNDF did not differ (40.8% uNDF; *p* = 0.12). Non-BMR genotypes contained greater concentrations of uNDF (*p* < 0.01) than BMR genotypes in corn (43.0 vs. 34.1% uNDF) and pearl millet (47.5 vs. 37.9% uNDF) but not in sorghum (41.2% uNDF). All forage types had similar degradation rates of pdNDF (2.3%/h; *p* = 0.57), and BMR genotypes had greater degradation rates of pdNDF than non-BMR genotypes (2.5 vs. 2.0%/h, respectively; *p* = 0.04). No interaction existed between forage type and genotype for the degradation rates of pdNDF (*p* = 0.78). The ERD of NDF differed among forage types (*p* = 0.05), being greatest for corn (22.9% NDF) and least for sorghum and pearl millet (19.5 and 19.3% NDF, respectively). Brown midrib genotypes had greater ERD of NDF than non-BMR genotypes (23.2 vs. 17.9% NDF, respectively; *p* < 0.01). No interaction existed between forage type and genotype (*p* = 0.51) for the ERD of NDF.

## 4. Discussion

The BMR phenotype in warm-season annual forages has been associated with a reduced lignin concentration compared to the non-BMR phenotype, and this reduced lignin concentration is attributed to a reduction in the synthesis of monolignols [19], the monomeric units for lignin synthesis. Several studies have reported greater NDF degradability for BMR forages than for non-BMR forages [5,6,7]. Based on the existing data, it may be assumed that BMR sorghum can be used as an alternative forage to non-BMR corn. Some advantages of replacing non-BMR corn with BMR sorghum are the lower expenditure in seed and the greater tolerance to drought stress [2,3,4]. However, a study from our laboratory suggested that expecting greater NDF degradability for BMR forages can be misleading when different forage types are compared. Specifically, Yang et al. fed high-producing dairy cows with diets containing either non-BMR corn silage or BMR sorghum silage, and observed a similar (56.7%) total tract NDF degradability. Furthermore, Yang et al. reported that BMR sorghum silage contained 11.6% ADL (NDF basis), whereas non-BMR corn silage contained 5.8% ADL (NDF basis) [8]. This observation indicates that forages containing the BMR mutation do not necessarily have the lowest ADL concentration (NDF basis) when comparing across forage types.

In line with the observations from Yang et al. [8], in the present study, although BMR corn had a lower ADL concentration than non-BMR corn (3.6 vs. 5.0% ADL), BMR sorghum had a lower ADL concentration than non-BMR sorghum (7.8 vs. 8.7% ADL), and BMR pearl millet had a lower ADL concentration than non-BMR pearl millet (5.2 vs. 7.3% ADL). Moreover, BMR sorghum and BMR pearl millet had greater ADL concentrations (NDF basis) than non-BMR corn (8.7, 7.3, and 5.0% ADL, respectively; Table 1). These data support our hypothesis that BMR genotypes may have lower ADL concentrations than non-BMR genotypes within forage types, but not when comparing across forage types.

Differences in NDF concentrations between BMR and non-BMR forages are not clear. For instance, Tine et al. and Coons et al. reported greater NDF concentrations (>2.0% NDF units) for non-BMR than for BMR corn hybrids, whereas Holt et al. and Ferraretto et al. reported greater NDF concentrations (>2.0% NDF units) for BMR than for non-BMR corn hybrids [20,21,22,23]. Several other studies reported similar NDF concentrations (<2.0% NDF units) for BMR and non-BMR corn silages [6,24,25,26,27,28,29]. In this study, in which we used two varieties of corn for each genotype, BMR and non-BMR whole-plants had similar NDF concentrations (42.2% NDF). Overall, data from this study and several previous studies suggest that differences in NDF concentrations between BMR and non-BMR genotypes are unlikely at the whole-plant level. 

Based on this study and several other studies, it is tempting to conclude that plants carrying the BMR mutation would not have lower NDF concentrations than plants not carrying the BMR mutation [6,24,25,26,27,28,29]. However, the nutritional composition at the tissue level (Table 1) challenges this possibility. Specifically, this study shows that plants carrying the BMR mutation have similar NDF concentrations than non-BMR plants in leaf blades, whereas plants carrying the BMR mutation have lower NDF concentrations in stem internodes than non-BMR plants. The latter is evident for corn (59.2 vs. 47.5% NDF) and pearl millet (58.8 vs. 50.5% NDF), but not for sorghum (54.2% NDF; Table 1). Based on our finding that the BMR mutation affects NDF concentration at the stem but not at the leaf level, it is prudent to suggest that the NDF concentration of whole-plant corn silage is only affected by the BMR mutation if there is an impact on the partition of leaves and stems of the whole plant.

The leaf-to-stem ratio has been used for decades as a broad indication of forage quality [30,31]. In this regard, because of the incomplete and non-uniform degradability of NDF, forages with greater proportions of leaves in their biomass are considered to be of better quality than forages with lesser proportions of leaves [32]. This concept relies partly on the fact that leaves have lower concentrations of NDF than stems [30]. Conversely, leaf blades mostly contained greater concentrations of NDF than stems in this study (Table 1). Though this may be considered unusual from a nutritional perspective, this observation is sound from a botanical perspective. The three warm-season grasses used in this study have a stem pith filled with parenchymal tissue that contains thin cell walls. As an example, unpublished data from our laboratory show that the pith from corn stem internodes contain a much lower NDF concentration than the cortex from the same corn stem internodes (35.4 vs. 52.3% NDF, respectively; Figure S1). In contrast, other grasses, such as barley (*Hordeum vulgare*), ryegrass (*Lolium multiflorum*), and switchgrass (*Panicum virgatum*), have hollow stems (Figure S2) that contain thick cell walls. This anatomical difference may explain why NDF concentration is much less sensitive to the leaf-to-stem ratio in certain warm-season grasses than in other cool-season grasses. Similarly, because the concentration of NDF in leaves is not affected by the BMR mutation, differences in NDF concentration at the whole-plant level might not be expected between BMR and non-BMR varieties.

Differences in the composition at the tissue level also explain the sensitivity of ADL concentration to the BMR mutation among forage types. In this study, BMR corn and pearl millet contained substantially less ADL (DM basis) than non-BMR corn and pearl millet (2.2 vs. 1.5% ADL and 3.7 vs. 2.6% ADL, respectively). Conversely, BMR sorghum contained marginally less ADL (DM basis) than non-BMR sorghum (4.3 vs. 3.9% ADL). The different sensitivities of ADL concentration among forage types can be attributed, at least in part, to the differences in NDF (DM basis) and ADL (NDF basis) concentrations of stem internodes.

As expected, at the whole-plant level, BMR varieties had a lower uNDF concentration (NDF basis) than non-BMR varieties, and corn had a lower uNDF concentration than sorghum and pearl millet (Figure 1). Furthermore, non-BMR corn had a lower uNDF concentration than BMR sorghum (25.1 vs. 31.3% uNDF), but similar uNDF to BMR pearl millet (24.1% uNDF). Altogether, these data support that, when comparing different forage types, there is no reason to assume that forages containing the BMR mutation in their genome should have the greatest NDF degradability. 

For leaf blades, pearl millet had the lowest uNDF concentration (NDF basis, Table 2) of all forage types, and BMR and non-BMR varieties of pearl millet had similar uNDF concentrations. Additionally, corn had a lower uNDF concentration than sorghum in both BMR (14.5 vs. 20.2%, respectively) and non-BMR (21.4 vs. 23.2%, respectively) varieties. From a non-quantitative visual appraisal, the midribs of corn and sorghum seemed to be proportionally larger than the midribs of pearl millet, and this anatomical difference might explain the differences in uNDF concentration in leaf blades among forage types. 

For stem internodes, no differences existed in uNDF concentration among forage types (NDF basis; Table 2), but BMR corn had a substantially lower uNDF concentration than non-BMR corn (34.1 vs. 43.0% uNDF), and BMR pearl millet had a substantially lower uNDF concentration than non-BMR pearl millet (47.5 vs. 37.9% uNDF). For sorghum, the BMR mutation did not affect the concentration of uNDF in the stem internodes. In this study, the lack of effect of the BMR mutation on uNDF concentration in sorghum is unexpected. Due to commercial availability, varieties of sorghum utilized in this study included a mixture of forage sorghum, sorghum × Sudan grass, and Sudan grass. It is possible that some varieties are more or less sensitive to the BMR genotype. Potential uNDF differences between BMR and non-BMR genotypes within the sorghum stem internodes could have been lost due to the variability within this study.

Regarding ruminal fiber degradation kinetics, the degradation rate of pdNDF did not differ among forage types at the whole-plant, leaf blade, or stem internode levels. BMR genotypes consistently had faster degradation rates by at least 0.2% units, with the greatest difference being at the whole-plant level. Despite degradation rate not being affected by forage type, there was a forage type effect on ERD at the whole-plant and stem internode level, such that corn had the greatest ERD, and sorghum and pearl millet had similar ERD (Table 2). In line with our hypothesis, these data support the existence of differences among forage types regarding ruminal degradation. The greater ERD of BMR varieties in all forage types, and the lack of interaction for ERD between forage type and genotype, also indicates that it cannot be assumed that forages containing the BMR mutation in their genome should have the greatest NDF degradability among different forage types.

In this study, the BMR genotypes had approximately 0.5% units greater CP concentrations within forage types at the whole-plant and stem internode levels. Differences in CP related to the BMR genotype are not consistent in the literature within and among forages. Some studies found that the BMR genotype increases CP concentration [7,24,33,34], while others did not observe a difference between BMR and non-BMR genotypes of the same forage types [35,36,37]. However, studies that did observe a difference only reported differences of, at most, 2.7% units. These data suggest that forages may be more or less sensitive to the BMR genotype regarding CP concentration, and that the BMR genotype may predominantly affect lignin concentrations and its subsequent relationships with fiber content and digestibility. 

## 5. Conclusions

Even though forages containing the BMR mutation may show lower lignin concentrations in the cell wall and greater fiber degradability than non-BMR forages of the same forage types, forages containing the BMR mutation do not always have the lowest lignin concentration and greatest fiber degradability when comparing different forage types. The responses of forage quality and degradability to the BMR mutation at the whole-plant level seem to be highly influenced by the partition of tissues, such as those of the leaves and stems.

## Figures and Tables

**Table 1:** Nutritional composition ^†^ of whole plants and plant tissues of warm-season annual grasses of different forage types (F) and genotypes ^‡^ (G).

	Corn		Sorghum		Pearl Millet			*p* <		
	CONV	BMR	CONV	BMR	CONV	BMR	SEM	F	G	F × G
*Whole Plant*										
CP, % DM	6.0	6.2	6.9	7.7	8.6	8.9	0.27	0.01	0.02	0.57
NDF, % DM	42.9	41.6	48.8	49.2	50.4	50.4	0.84	0.01	0.89	0.47
ADF, % DM	24.1	22.6	30.1	30.4	29.8	28.5	0.45	0.01	0.02	0.10
ADL, % DM	2.2 ^d^	1.5 ^e^	4.3 ^a^	3.9 ^b^	3.7 ^b^	2.6 ^c^	0.11	0.01	0.01	0.01
ADL, % NDF	5.0 ^c^	3.6 ^d^	8.7 ^a^	7.8 ^b^	7.3 ^b^	5.2 ^c^	0.21	0.01	0.01	0.02
*Leaf Blades*										
CP, % DM	12.0	11.2	13.3	14.1	12.0	12.4	0.48	0.01	0.91	0.28
NDF, % DM	59.1	59.3	60.3	59.3	55.1	56.5	0.71	0.01	0.75	0.29
ADF, % DM	34.6	33.8	34.3	33.2	31.8	31.2	0.61	0.02	0.12	0.93
ADL, % DM	4.9 ^a^	3.9 ^b^	4.3 ^b^	4.0 ^b^	4.3 ^b^	2.9 ^c^	0.17	0.01	0.01	0.04
ADL, % NDF	8.3 ^a^	6.6 ^d^	7.1 ^bc^	6.8 ^cd^	7.9 ^ab^	5.1 ^e^	0.34	0.17	0.01	0.02
*Stem Internodes*										
CP, % DM	1.7	1.8	2.1	2.7	2.2	3.0	0.24	0.01	0.01	0.25
NDF, % DM	59.2 ^a^	47.5 ^d^	54.5 ^bc^	53.8 ^c^	58.8 ^ab^	50.5 ^cd^	1.55	0.32	0.01	0.01
ADF, % DM	40.7 ^a^	30.8 ^d^	37.3 ^b^	36.7 ^b^	38.8 ^ab^	32.7 ^d^	1.16	0.37	0.01	0.01
ADL, % DM	4.8 ^a^	2.2 ^c^	4.8 ^a^	4.1 ^b^	5.1 ^a^	2.4 ^c^	0.25	0.01	0.01	0.01
ADL, % NDF	8.0 ^a b^	4.4 ^c^	8.6 ^a^	7.1 ^b^	8.7 ^a^	4.9 ^c^	0.34	0.01	0.01	0.01

Different superscripts in the same row indicate a significant difference (*p* < 0.05). ^†^ DM = dry matter; CP = crude protein; NDF = neutral detergent fiber; ADF = acid detergent fiber; ADL = acid detergent lignin; ^‡^ CONV = conventional (or non-BMR); BMR = brown midrib.

**Table 2:** Fiber degradation kinetic parameters ^†^ for whole plants and plant tissues of warm-season annual grasses of different forage types (F) and genotypes ^‡^ (G).

	Corn		Sorghum		Pearl Millet			*p* <		
	CONV	BMR	CONV	BMR	CONV	BMR	SEM	F	G	F × G
*Whole Plant*										
uNDF, % NDF	25.1	18.2	36.3	31.3	34.4	24.1	1.3	0.01	0.01	0.13
pdNDF, % NDF	74.9	81.8	63.7	68.7	65.6	75.9	1.3	0.01	0.01	0.13
kd, %/h	2.5	3.0	2.4	3.6	2.3	2.7	0.3	0.37	0.01	0.33
ERD, % NDF	28.5	34.4	23.6	30.3	23.9	30.2	1.4	0.01	0.01	0.96
*Leaf Blades*										
uNDF, % NDF	21.4 ^ab^	14.5 ^c^	23.3 ^a^	20.2 ^b^	16.8 ^c^	15.2 ^c^	0.9	0.01	0.01	0.02
pdNDF, % NDF	78.6 ^bc^	85.5 ^a^	76.7 ^c^	79.8 ^b^	83.2 ^a^	84.8 ^a^	0.9	0.01	0.01	0.02
kd, %/h	2.7	2.9	2.7	2.9	2.6	2.9	0.2	0.86	0.08	0.96
ERD, % NDF	31.2	35.1	30.1	33.2	32.3	34.2	1.4	0.28	0.02	0.80
*Stem Internodes*										
uNDF, % NDF	43.0 ^abc^	34.1 ^d^	40.6 ^bc^	41.8 ^abc^	47.5 ^a^	37.9 ^cd^	2.2	0.12	0.01	0.02
pdNDF, % NDF	57.0 ^bcd^	65.9 ^a^	59.4 ^bc^	58.2 ^bcd^	52.5 ^d^	62.1 ^ab^	2.2	0.12	0.01	0.02
kd, %/h	2.2	2.7	1.9	2.7	1.9	2.2	0.3	0.57	0.04	0.78
ERD, % NDF	19.7	26.1	17.8	21.2	16.3	22.3	1.6	0.05	0.01	0.51

Different superscripts in the same row indicate significant difference (*p* < 0.05). ^†^ NDF = neutral detergent fiber; uNDF = undegraded neutral detergent fiber (after 240 h of fermentation); pdNDF = potentially degradable neutral detergent fiber; kd = degradation rate of pdNDF; ERD = effective ruminal degradation of NDF; ^‡^ CONV = conventional (or non-BMR); BMR = brown midrib.

## Data Availability

The data are not publicly available due to commercial restrictions.

## Supplementary Materials

The following supporting information can be downloaded at: https://www.mdpi.com/article/10.3390/ani12192536/s1. Figure S1: Neutral detergent fiber concentration in the inner pith and outer cortex of corn internodes; Figure S2: Cross sections of stems from corn (A) and switchgrass (B) depicting filled and hollow stems, respectively.

## Abbreviations

ADF, acid detergent fiber; ADL, acid detergent lignin; BMR, brown midrib; CP, crude protein; df, degrees of freedom; DM, dry matter; ERD, effective ruminal degradation; ISNDFD, in situ NDF degradability; kd, degradation rate; kp, passage rate; NDF, neutral detergent fiber; NDFD, NDF degradability; pdNDF, potentially degradable NDF; uNDF, undegraded NDF.
