# Supplementary material for: Ruminal Fiber Degradation Kinetics within and among Warm-Season Annual Grasses as Affected by the Brown Midrib Mutation
Source: Animals (Basel). 2022 Sep 22;12(19):2536. doi: 10.3390/ani12192536 (PMC9558941; doi:10.3390/ani12192536)
Supplement: Supplementary file 1 [file animals-12-02536-s001.zip › animals-1877111-supplementary.pdf]

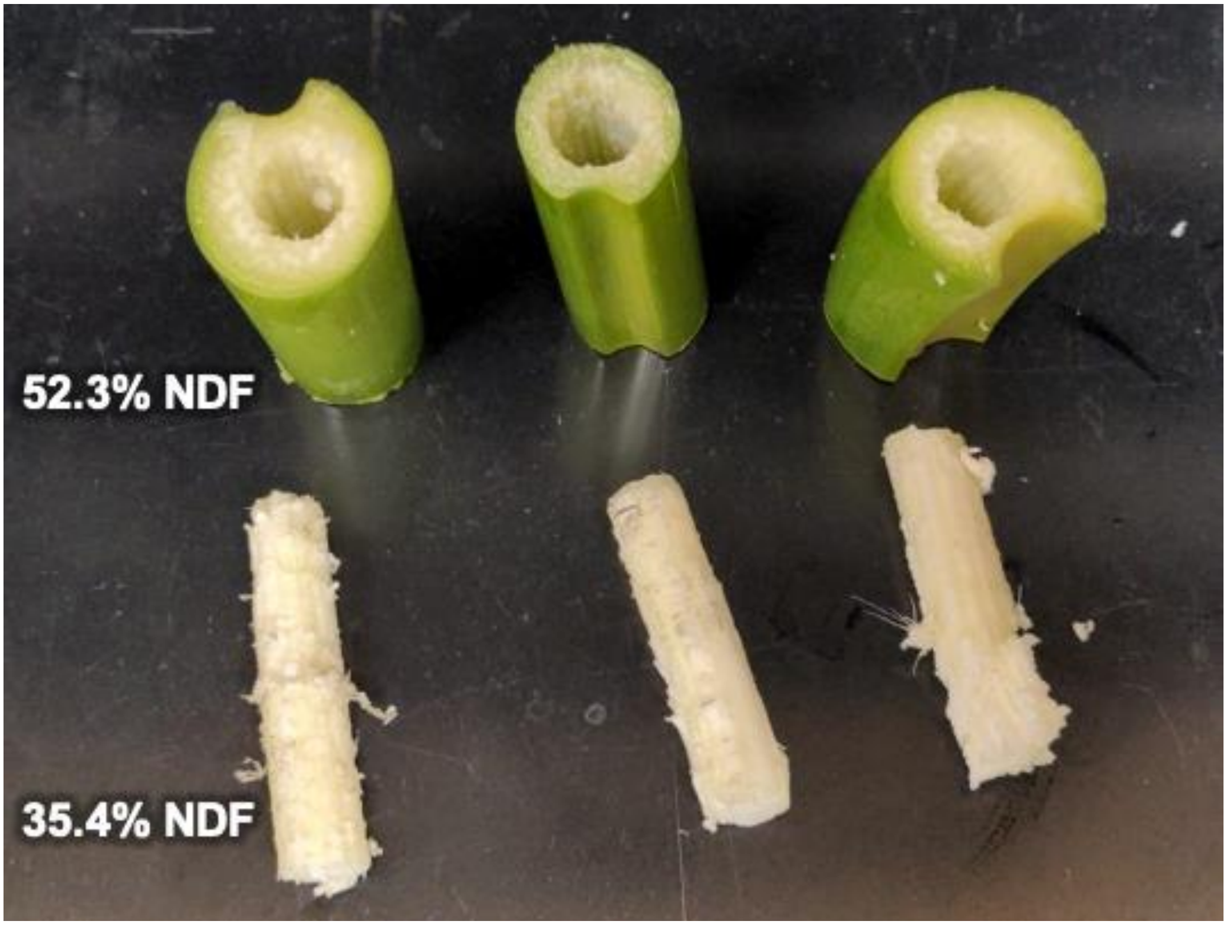

Figure S1. Neutral detergent fiber concentration in the inner pith and outer cortex of corn internodes.

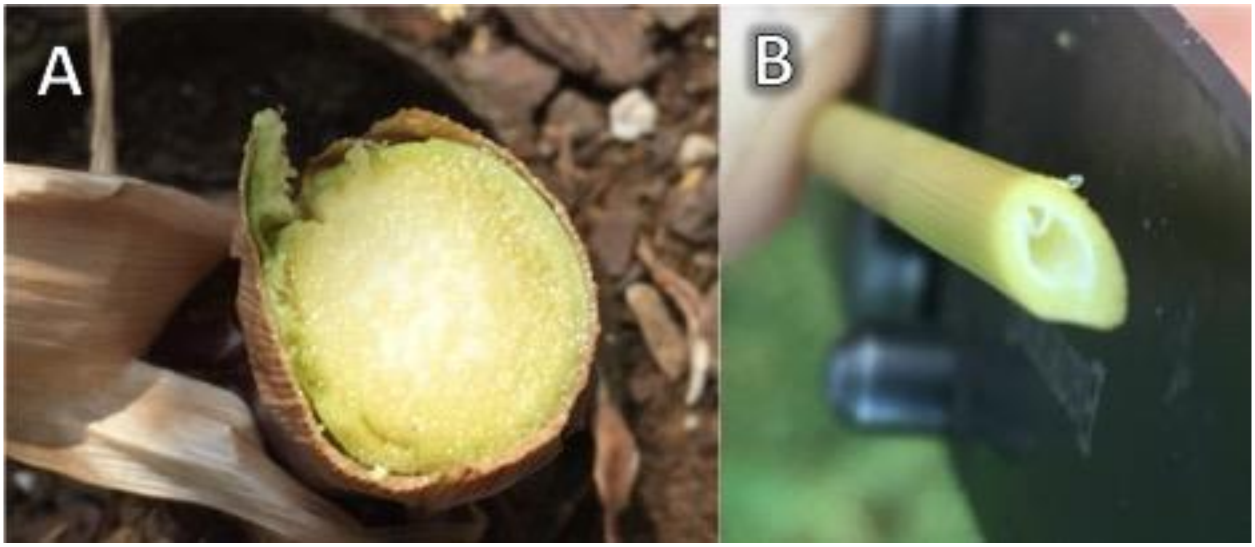

Figure S2. Cross sections of stems from corn (A) and switchgrass (B) depicting filled and hollow stems, respectively.
